# Structural Characterization and Immunoenhancing Properties of Polysaccharide CPTM-P1 from *Taxus media*

**DOI:** 10.3390/molecules29061370

**Published:** 2024-03-19

**Authors:** Jiangtao Fan, Xiong Huang, Mengke Dou, Shuqin Tang, Gang Wang, Yijun Fan, Aoxue Luo, Gang Wang, Yong Wang

**Affiliations:** 1 National Forestry and Grassland Southwest Engineering Technology Research Centre of Taxus, Sichuan Agricultural University, Dujiangyan 611800, China; jiangtao_fan@163.com (J.F.); huangdoctor226@163.com (X.H.); dmk15836@163.com (M.D.); tangshuqin2021@163.com (S.T.); wg@cdut.edu.cn (G.W.); yijfan@163.com (Y.F.); aoxueluo@sina.com (A.L.); 2 College of Forestry, Sichuan Agricultural University, Chengdu 611130, China; 3 College of Geography and Planning, Chengdu University of Technology, Chengdu 611130, China

**Keywords:** *Taxus media*, polysaccharide, structural characterization, immunoenhancing properties

## Abstract

Polysaccharides extracted from *Taxus media* hrough an aqueous method were further refined by removing proteins via the Sevag technique and purified by dialysis. The separation of these polysaccharides was accomplished using a DEAE-cellulose chromatog-raphy column, yielding two distinct fractions, named CPTM-P1 and CPTM-P2. Notably, CPTM-P1 emerged as the primary polysaccharide component within *Taxus media*. Consequently, a comprehensive analysis focusing exclusively on CPTM-P1 was undertaken. The molecular weight of CPTM-P1 was established through gel permeation chromatography (GPC), and its monosaccharide composition was deciphered using HPLC-MS. The structure was further elucidated through nuclear magnetic resonance (NMR) spectroscopy. The molecular weight of CPTM-P1 was determined to be 968.7 kDa. The monosaccharide composition consisted of galactose (Gal), arabinose (Ara), galacturonic acid (Gal-UA), glucose (Glc), rhamnose (Rha), xylose (Xyl), mannose (Man), fucose (Fuc), glucuronic acid (Glc-UA), and ribose (Rib). The proportional distribution of these components was 30.53%, 22.00%, 5.63%, 11.67%, 11.93%, 1.69%, 8.50%, 1.23%, 5.63%, and 1.17%, respectively. This confirmed CPTM-P1 as an acidic heteropolysaccharide with a glycuronic acid backbone. Moreover, CPTM-P1 showed immunoenhancing properties, effectively augmenting the secretion of nitric oxide and cytokines (TNF-α, IL-1β, and IL-6). Additionally, it significantly enhances the phagocytic capacity of RAW264.7 cells. These findings underscore the potential application of these polysaccharides in functional foods and pharmaceuticals, providing a solid scientific basis for further exploration and utilization of *Taxus media* polysaccharides.

## 1. Introduction

Polysaccharides, complex biopolymers consisting of 10 or more monosaccharides linked by glycosidic bonds, stand as some of nature’s most prevalent molecules [1]. Research on polysaccharides is expanding due to their potent anti-tumor [2], hypoglycemic [3], antioxidant [4], anti-allergic [5], and immunomodulatory [6] effects, and their capacity to shield cells from inflammatory cytokines [7]. The evaluation of polysaccharides’ immunomodulatory capabilities often involves measuring cytokine levels released by macrophages following polysaccharide stimulation [8,9]. Research has underscored polysaccharides’ capacity to stimulate macrophages, enhance phagocytosis, and trigger a broad spectrum of cytokine production, including interleukins (IL-1β, IL-6, IL-8), tumor necrosis factor (TNF-α), and nitric oxide (NO). Specifically, IL-1β is produced by activated immune cells, playing a crucial role in driving immune and inflammatory responses, including fever induction, immune cell activation, and inflammation mediation. IL-6, a key marker of acute inflammation, is secreted by various cells and is pivotal in immune regulation, inflammation, cell growth, and differentiation. IL-8, primarily released by macrophages and endothelial cells, acts as a chemoattractant that directs white blood cells, especially neutrophils, to inflammation sites, playing a significant role in both inflammation and immune regulation [10,11]. Consequently, these actions reinforce the organism’s immune response [10,11,12,13]. Certain plant polysaccharides are capable of augmenting the secretion of nitric oxide (NO) and cytokine synthesis of cytokines, thereby enhancing the efficacy of macrophages in combating pathogenic microorganisms and tumors [14]. Nitric oxide is a key effector molecule produced by macrophages, which upon activation, release various chemotactic factors and cytokines crucial for activating immune responses and modulating the immune system [15]. Activated macrophages play an important role in TNF-α generating, serving as a pivotal cytokine in anti-tumor immune responses and an essential indicator of macrophage activity [16]. IL-6 plays a versatile role in immune defense, underscoring the complex interactions within the immune system [17].

The plant kingdom boasts a rich diversity of polysaccharides. Previous studies have successfully isolated polysaccharides from a variety of plants, including *Ziziphus jujuba* [18], *Astragalus membranaceus* [19], *Panax ginseng* [4], and persimmon [7], demonstrating their potent immunomodulatory and outstanding antioxidant activities in vitro. In the genus *Taxus*, significant research efforts have yielded notable findings. Complex water-soluble polysaccharide (T1) and a singular polysaccharide component (CPTC-2) were isolated from the branches and leaves of *Taxus chinensis* [20,21]. Additionally, TCFPs, a polysaccharide from *T. chinensis* fruits, exhibited significant tumor growth inhibition [22]. Two novel water-soluble polysaccharides with anticancer properties, TMP70S-1 and TMP70W, were identified in the branches and leaves of *Taxus yunnanensis* [23,24]. *Taxus cuspidata* yielded four polysaccharides (Pe1, Pe2, Pe3, and Pe4), with Pe4 exhibiting the strongest anti-tumor and glucosidase inhibitory properties [25]. Moreover, these studies also revealed that *Taxus* polysaccharides exhibit minimal toxic side effects and potent pharmacological effects. They can reduce the dosage of chemotherapy drugs, thereby lowering the toxic side effects of chemotherapy. As a result, they present themselves as promising adjunctive medications for clinical chemotherapy in cancer patients, showcasing excellent prospects for medicinal use [22,23,24,25]. Despite these advancements, research into the unique polysaccharides found in *Taxus media* branches and leaves, particularly their immunoenhancing properties, remains unpublished, presenting a gap in the development of therapeutic polysaccharides.

This study focused on analyzing the molecular weight, monosaccharide composition, and glycan chain structure of CPTM-P1. Additionally, it aimed to explore its immunoenhancing properties using RAW 264.7 macrophage cells. This dual approach seeks to deepen our understanding of CPTM-P1′s structural and biological characteristics.

## 2. Results and Discussion

### 2.1. Isolation and Purification of Polysaccharides from T. media

**Figure 1:**
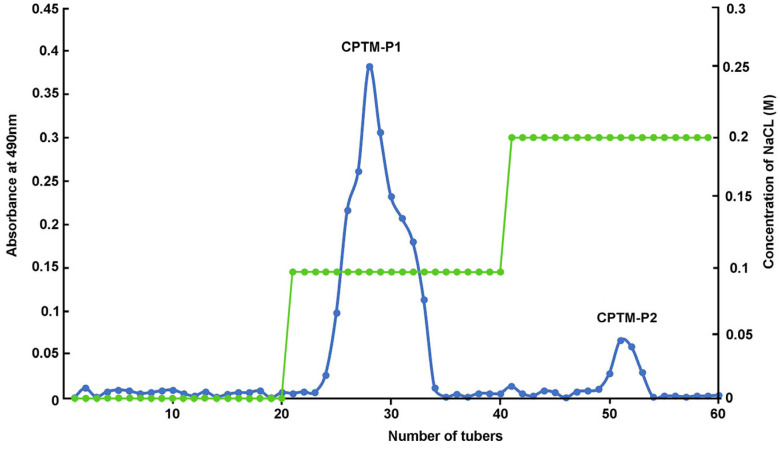
Chromatography of eluted crude polysaccharide (CPTM−CP) on a DEAE–cellulose column (26 mm × 300 mm). CPTM−P1 eluted with 0.1 M NaCl; CPTM-P2 eluted with 0.2 M NaCl.

Through a process of staged elution with a NaCl solution, two distinct polysaccharide components were obtained, named CPTM-P1 and CPTM-P2, as illustrated in Figure 1. CPTM-P1 was isolated using a wash with 0.1 M NaCl, while CPTM-P2 was separated using 0.2 M NaCl. Among these components, CPTM-P1 constitutes the largest fraction and is identified as the predominant polysaccharide in *T. media*. Consequently, the focus of this study will be primarily on CPTM-P1, aiming to delve into the structural characteristics and immuno-enhancing properties of this polysaccharide. CPTM-CP, the crude polysaccharide extract from *T. media*, serves as the basis for isolating these components.

### 2.2. Analysis of Structural Characteristics of Polysaccharides

#### 2.2.1. Molecular Weight Analysis

**Figure 2:**
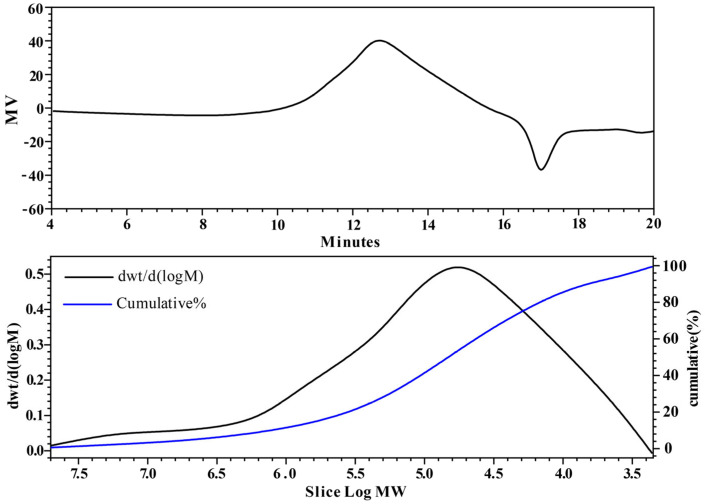
Analysis of CPTM−P1 polysaccharide by GPC.

The molecular weight (Mw) of polysaccharides plays a critical role in defining their properties, which largely depend on their molecular dimensions [26]. Typically, the distribution of Mw is represented by an average value. The Mw and purity of polysaccharide samples were determined using Gel Permeation Chromatography (GPC) and analyzed with Empower software (v3.8.0), as shown in Figure 2. This analysis revealed that CPTM-P1 has a molecular weight of 968.7 kDa, a polydispersity index (PDI) of 1.126, and a purity of 79%. It has been demonstrated that polysaccharides with different molecular weights possess distinct biological functions. For example, fucoidan with a lower molecular weight (<50 kDa) exhibits stronger immunomodulatory effects compared to those with a higher molecular weight (>100 kDa) [27,28]. Similarly, the high molecular weight polysaccharide from persimmon, DK-H (>345 kDa), shows enhanced physiological effects, including antioxidative, anti-inflammatory, and anti-wrinkle properties, unlike its low molecular weight counterpart, DK-L (<1 kDa) [7].

Research has consistently shown that polysaccharides with higher molecular weights tend to have greater pharmacological efficacy than those with lower molecular weights [29,30]. However, it is imperative to acknowledge that certain low molecular weight polysaccharides (<10 or 30 kDa) have been reported to lack biological activity under specific circumstances [31]. The polysaccharide CPTM-P1 (968.7 kDa) investigated in this study exhibits a greater molecular weight in comparison to the *T. chinensis* polysaccharide CPTC-2 (73.53 kDa), and the *T. yunnanensis* polysaccharides TMP70W (36.94 kDa) and TMP70S-1 (17.37 kDa). This molecular weight difference suggests the potential for heightened biological activity in CPTM-P1 [20,23,24]. In summary, the molecular weight of polysaccharides assumes an important role in shaping their biological activities.

#### 2.2.2. Infrared Spectrum Analysis

**Figure 3:**
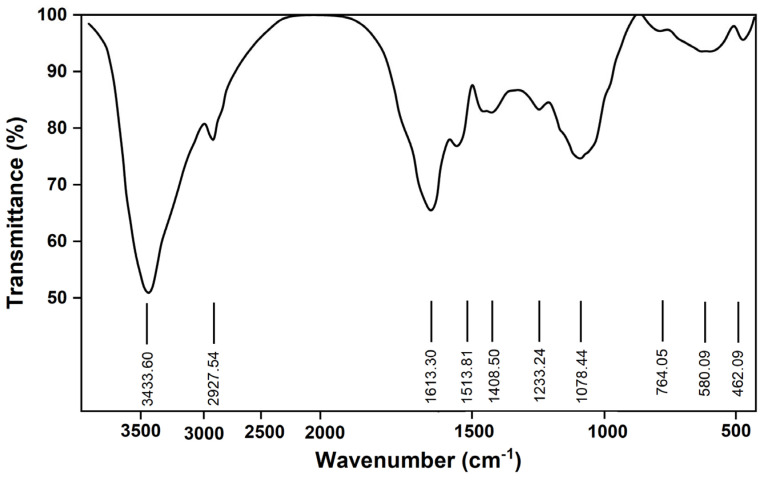
FTIR analysis of CPTM−P1 polysaccharide from *T. media*.

Fourier-transform infrared (FT-IR) spectroscopy is an essential technique in organic and polymer chemistry, allowing for the quantitative analysis of specific com-pounds through infrared spectroscopy [32]. This method was employed to examine the functional groups and sugar chain structure of CPTM-P1. The FT-IR spectrum of CPTM-P1, depicted in Figure 3, features a pronounced broad peak between 3600−3200 cm^−1^, indicative of the stretching vibrations of hydroxyl (–OH) groups within the sugar units. This observation suggests the presence of both intermolecular and intramolecular hydrogen bonds. The weak C−H absorption seen between 3000–2800 cm^−1^, coupled with an absorption peak in the 1400−1200 cm^−1^ range signaling C−H bending vibrations, confirms that CPTM-P1 is a polysaccharide. Typically, saccharide hydrates display characteristic absorption peaks within 1665−1635 cm^−1^, a feature also observed in the CPTM-P1 polysaccharide spectrum. Moreover, distinct peaks at 1223 cm^−1^ and 1078 cm^−1^ signify the presence of pyran rings in CPTM-P1’s structure. Furthermore, a peak at 746 cm^−1^ delineates the α-configuration of this poly-saccharide, providing insights into its molecular architecture.

#### 2.2.3. Monosaccharide Analysis

Ten different monosaccharides were identified in CPTM-P1. Which included Fuc, Ara, Rha, Gal, Glc, Xyl, Man, Rib, Gal-UA, and Glc-UA. Fru, Gul-UA, and Man-UA were not detected (Table 1). Notably, Gal emerged as the most predominant constituent. Following Gal, Gal-UA exhibited a content of 27.42 mg/g, while Ara demonstrated a concentration of 26.24 mg/g, and Rha exhibited a measure of 14.23 mg/g. Similarly, Glc and Man registered values of 13.92 mg/g and 10.14 mg/g, respectively. Conversely, the remaining monosaccharides were characterized by diminutive peak areas, indicating the relatively marginal abundance of their respective CPTM-P1 polysaccharide constituents (less than 10 mg/g).

Research has established a strong correlation between the structural features and pharmacological activities of polysaccharides [33]. Complex carbohydrates include galacturonic and gluturonic acids in their structure [34]. Findings suggest that Gal-UA may have anti-inflammatory effects and play a role in immune response regulation. Additionally, the amount of Ara in polysaccharides is positively associated with their immune activity [35]. CPTM-P1 contains elevated levels of both Gal-UA and Ara, which could potentially account for the observed immune activity of CPTM-P1. The presence of glyoxylate residues has the capacity to alter the physicochemical properties and solubility of associated polysaccharide couplings, thereby influencing the activity of plant polysaccharides [36]. Fractions characterized by a higher glyoxalate content demonstrate heightened antioxidant activity. Specifically, ZSP3c and ZSP4b, featuring the highest glyoxalate content among date polysaccharide fractions, exhibit the most robust in vitro antioxidant activity [37]. Galactose also influences the immune-modulating activity of polysaccharides; research has identified galactose in *A. membranaceus* polysaccharides as the primary factor responsible for immune-regulatory activity [38]. Another study has shown that polysaccharides with a higher galactose (Gal) content exhibit superior immune-modulating activity [39]. CPTM-P1 also has a significant galactose content, suggesting its activity may be closely related to its glyoxalate and galactose levels. Furthermore, pumpkin polysaccharides, which include glucuronic acid, show that higher concentrations of glucuronic acid correlate with increased biological activity. Among pumpkin polysaccharides, WPP2, which has the highest glyoxalate content, demonstrated superior activity compared to WPP0, WPP1, and WPP3 [40]. However, CPTM-P1 contains an equal amount of galacturonic and glucuronic acids, possibly contributing to its immunomodulatory properties. Polysaccharides in plants exist in a variety of forms, such as pectic polysaccharides. Pectic polysaccharides usually contain components such as Galactose (Gal), Arabinose (Ara), Galacturonic acid (Gal-UA), etc., so, based on the composition of the monosaccharides, we hypothesized that the CPTM-P1 polysaccharides might belong to the category of pectic polysaccharides. In plant cell walls, various substances, including cellulose, hemicellulose, and pectin, are present [41]. Although our study’s crude polysaccharide extract includes a mix of cellulose, hemi-cellulose, and starch, the active polysaccharides isolated and purified did not react with Congo red for cellulose or iodine for starch, excluding them as cellulose, pectin, or starch components.

#### 2.2.4. Nuclear Magnetic Resonance (NMR) Analysis

**Figure 4:**
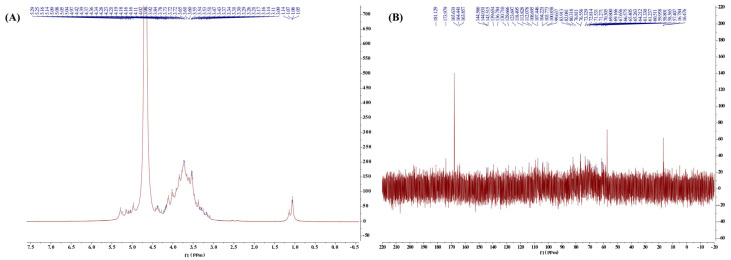
NMR analysis results. (**A**) is the ^1^H NMR of CPTM−P1, (**B**) is ^13^C NMR of CPTM−P1.

To delve deeper into the molecular architecture of CPTM-P1, a detailed nuclear magnetic resonance (NMR) spectroscopy analysis was performed. Figure 4 shows the ^1^HNMR and ^13^C NMR spectra of CPTM-P1. In the ^1^H NMR spectrum, most of the signals appeared in the range of 4.8–5.5 ppm. Typically, an anomeric signal above 4.9 ppm indicates an α-configuration, whereas a signal falling below 4.9 ppm indicates a *β*-configuration [42,43]. Evidently, the allosteric signal in CPTM-P1′s 1H spectrum is located around 5.0 ppm (Figure 4A). Therefore, CPTM-P1 has an *α*-configuration. In the ^13^C spectrum, resonances within 50–85 ppm correspond to carbons at positions C-2 to C-6 (Figure 4B). Characteristic signals arising from the carboxyl groups are discernible within the spectral region of 165–180 ppm. Specifically, the distinct peaks at 177.848 ppm and 167.858 ppm in CPTM-P1′s carbon spectrum indicate –CO signals inherent to the –COOH moiety. This resonance pattern serves to confirm the presence of uronic acids within the molecular composition of CPTM-P1; a deduction further substantiated by the findings of HPLC-MS analysis. It is well known that the type of glycosidic linkage plays a crucial role in determining the immunomodulatory activity of polysaccharides [44]. For example, the anti-inflammatory efficacy of *Poria cocos* polysaccharides has been linked to a 1,3-α-d-Galp backbone and 1,6-α-d-Galp branches [45]. Although the sugar chains in CPTM-P1 exhibit similarities, future research must investigate the specific linkage patterns within CPTM-P1 and their influence on immunomodulatory activity.

Drawing upon the existing literature [46,47,48], our deductions ascertain the principal occurrence of the indicated glycan chains within the constitution of CPTM-P1 (refer to Table 2). However, due to the constraints imposed by the finite data yielded by NMR, certain glycan chains characterized by lower prevalence could not be definitively resolved.

#### 2.2.5. SEM Analysis Results

**Figure 5:**
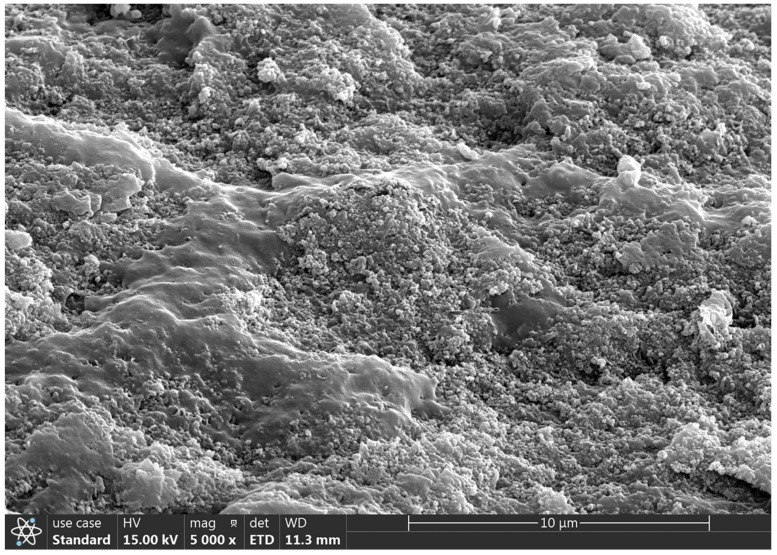
Morphology observation of CPTM−P1 polysaccharide under SEM.

Utilizing Scanning Electron Microscopy (SEM) enables the acquisition of high-resolution data on surface topography, providing detailed insights into the particle size, shape, and surface characteristics of polysaccharides. This information is pivotal for understanding the spatial arrangement and structural details of these biomolecules [39,40]. Consequently, SEM was used to examine the structure and spatial organization of the CPTM-P1 surface. The SEM images (Figure 5) reveal that the CPTM-P1 polysaccharide surface is predominantly smooth and features convex, semi-circular particles within a notable layered structure. This observation suggests that the polysaccharide’s branched monomers are intricately intertwined, predominantly existing in states of aggregation and coiling.

### 2.3. Effect of CPTM-P1 on RAW264.7 Cell Immunoreactivity

#### 2.3.1. Effect of CPTM-P1 on RAW264.7 Cell Proliferation

**Figure 6:**
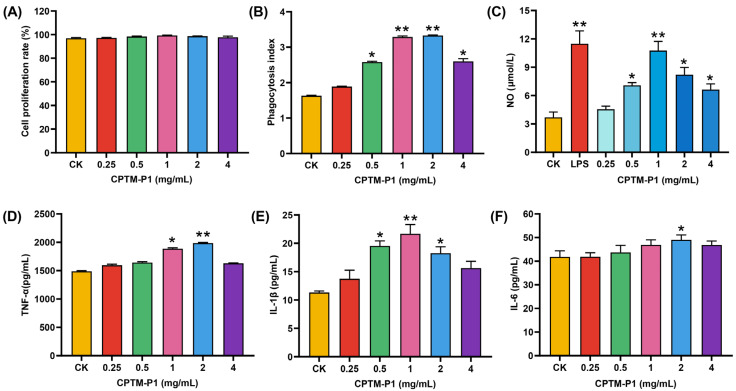
(**A**) Effects of different concentrations of CPTM−P1 on the proliferation of RAW264.7 cells; (**B**) effect of CPTM−P1 on phagocytosis of RAW264.7 cells; (**C**) detection of NO release from RAW264.7 cells in response to CPTM−P1, the concentration of LPS was 0.5 μg/mL. ELISA detection of (**D**) TNF-α, (**E**) IL-1β and (**F**) IL-6. * indicated *p* < 0.05; ** indicated *p* < 0.01.

The effects of CPTM-P1 on RAW264.7 cells proliferation are shown in Figure 6A. When compared to the control group (CK), the stimulation of RAW264.7 cell proliferation by CPTM-P1 exhibited a trend of increase in line with rising concentrations, peaking at 1.0 mg/mL. However, there were no statistically significant differences between the experimental groups at varying concentrations and the control group (*p* > 0.05). This finding supports the conclusion that CPTM-P1, within a concentration range up to 4.0 mg/mL, does not show cytotoxic effects on RAW264.7 cells. The optimal conditions for polysaccharide intervention are often established by evaluating their effects on cell viability [49]. The investigation revealed that within the concentration range of 0.25–4 mg/mL, there existed a direct relationship between the concentration and the proliferation rate of RAW 264.7 cells, reaching its zenith at 1.0 mg/mL. Notably, even at the highest concentration of 4 mg/mL, no statistically significant distinction was discerned between the control group and the experimental group. These results indicate that, within a specific concentration range, CPTM-P1 does not induce cytotoxic effects on RAW 264.7 cells.

#### 2.3.2. Effect of CPTM-P1 on RAW264.7 Cell Phagocytosis

Data on the impact of different polysaccharide concentrations on RAW264.7 cell phagocytosis is presented in Figure 6B. The findings underscore that all concentrations of the polysaccharide CPTM-P1 elicited a discernible enhancement in the phagocytic efficacy of RAW264.7 cells. The cellular phagocytic activity reached its zenith at a concentration of 2.0 mg/mL (*p* < 0.01), followed by a modest decline in phagocytic activity.

#### 2.3.3. CPTM-P1 Effect on NO, TNF-a, IL-1β, and IL-6 Production

Activated macrophages perform dual functions: they secrete cytokines and synthesize nitric oxide (NO). In this research, we further explored NO secretion by RAW264.7 cells activated with the polysaccharide CPTM-P1 and LPS, as shown in Figure 6C. Notably, LPS significantly elevated NO levels in RAW264.7 cells. The introduction of varying concentrations of CPTM-P1 led to increase NO secretion, with the effect intensifying at higher polysaccharide concentrations. This increase was most significant at 1.0 mg/mL, equating the effect to that of the LPS positive control. Beyond this concentration, a slight decline in NO production was noted.

The study also examined the effects of CPTM-P1 on the secretion of TNF-α, IL-1β, and IL-6 by RAW264.7 cells, considering the critical roles these cytokines play in inter-cellular communication, immune cell regulation, and inflammatory responses (Figure 6D–F). The data reveal that varying CPTM-P1 concentrations differently influenced TNF-α, IL-1β, and IL-6 levels in the cell culture supernatant. A pattern emerged where cytokine concentrations initially increased and then decreased, with TNF-α and IL-6 peaking at 2.0 mg/mL (*p* < 0.05), and IL-1β peaking at 1.0 mg/mL (*p* < 0.05). Past these peaks, a slight decrease in cytokine levels was observed. These results collectively suggest that CPTM-P1 enhances the immunomodulatory capacity of RAW264.7 cells by stimulating the secretion of TNF-α, IL-1β, and IL-6.

One study shows that lipopolysaccharides (LPS) trigger immune stresscan incite immune stress by stimulating host immune cells to produce nitric oxide (NO) and cytokines, including tumor necrosis factor-alpha (TNF-α), interleukin-1β (IL-1β), and interleukin-6 (IL-6) [50]. Due to their capability to release NO, activated RAW264.7 cells are frequently used to evaluate the immunomodulatory activity of compounds [27]. Thus, LPS-treated RAW 264.7 cells served as a positive control for assessing the immunomodulatory potential of CPTM-P1. Significantly, CPTM-P1 induced NO release from RAW 264.7 cells and increased levels of TNF-α, IL-1β, and IL-6, indicating that CPTM-P1 can activate macrophages and elicit immune responses akin to LPS. This aligns with observations of polysaccharides from sweet cherry, berries, *Cucurbita moschata*, and lavender, which also modulate immune function by activating macro-phages [51,52,53,54]. While CPTM-P1’s immunomodulatory effects are confirmed, further investigation into its mechanistic pathways is necessary.

### 2.4. Endotoxin Contamination

**Figure 7:**
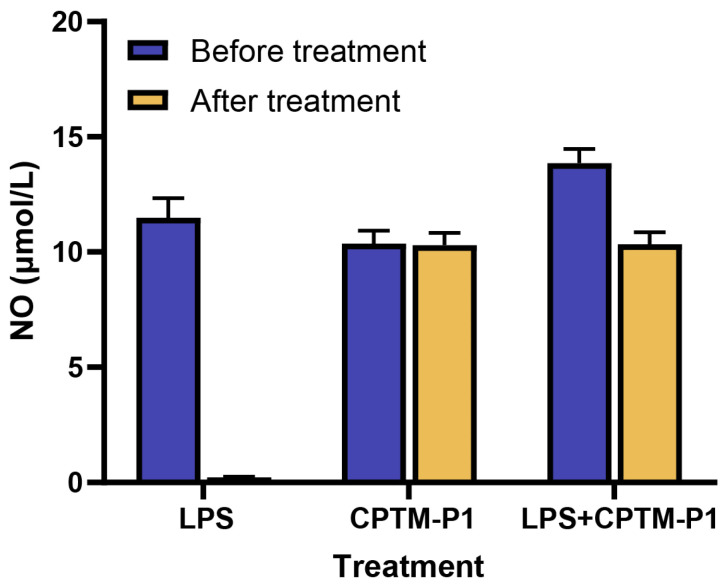
Effects of polymyxin B treatment. LPS (0.5 μg/mL), CPTM−P1 (1 mg/mL), or an LPS (0.5 μg/mL) and CPTM−P1 (1 mg/mL) mixture was added to the polymyxin B-affinity column, incubated overnight at 4 °C, eluted from the column by centrifugation, and then added to the cultures of RAW264.7 cells (final, 1:80 dilution) for 2 days. The amounts of nitric oxide were measured using a Griess reagent.

Extracting polysaccharides from plants carries a risk of endotoxin contamination, which can falsely enhance immune stimulation assays due to the potent macrophage activation by endotoxins [55,56]. To confirm that CPTM-P1’s immunomodulatory effects were not due to endotoxin contamination, we treated CPTM-P1 with polymyxin B and evaluated its activity in RAW264.7 cells. As depicted in Figure 7, the solution of lipopolysaccharide (LPS) (0.5 μg/mL), after passing through a polymyxin B affinity column, exhibited a nearly complete reduction in nitric oxide-induced activity. This suggests that the polymyxin B affinity column effectively absorbed almost all of the LPS. However, the CPTM-P1 solution (1 mg/mL) passed through the polymyxin B affinity column did not exhibit a significant reduction in nitric oxide-induced activity. To eliminate the potential interference of unknown substances in the CPTM-P1 solution with the formation of the polymyxin B complex, CPTM-P1 was mixed with LPS, passed through a polymyxin B affinity column, and then assessed for nitric oxide-induced activity in RAW264.7 cells. The results, as shown in Figure 7, indicate that the column effectively eliminated LPS from the mixture, confirming that CPTM-P1’s immunomodulatory effects are not due to endotoxin contamination.

## 3. Materials and Methods

### 3.1. Materials and Chemicals

Branches and leaves of *Taxus media* were collected in November 2022 from Xudu Garden, Dujiangyan, Sichuan, China, and were authenticated by Professor Xiaohong Chen from Sichuan Agricultural University. The specimens were archived at the National Forestry and Grassland Administration Southwest Engineering Technology Research Center for Taxus at Sichuan Agricultural University. The MTT kit, along with mouse TNF-α, IL-1β, and IL-6 ELISA kits, were acquired from Beijing Solabao (Beijing, China). RPMI 1640 medium and phosphate-buffered saline (PBS) for cell immunoreactivity assays were sourced from Thermo Fisher Scientific (Waltham, MA, USA), while trypsin and penicillin were obtained from Wistent (Saint-Jean-Baptiste, QC, Canada). Fetal bovine serum (FBS) was procured from Lanzhou Bailing Company (Lanzhou, China). DEAE–cellulose was purchased from (Sigma Chemical, St. Louis, MO, USA). Kormas Brilliant Blue G-250 and Trifluoroacetic acid (TFA) were purchased from (Chengdu Kelong Chemical Company, Chengdu, China). The Congo red reagent and iodine solution were purchased from Beijing Solebo. Monosaccharide standards including fucose (Fuc), arabinose (Ara), rhamnose (Rha), galactose (Gal), glucose (Glc), xylose (Xyl), mannose (Man), fructose (Fru), ribose (Rib), galacturonic acid (Gal-UA), glucuronic acid (Glc-UA), mannuronic acid (Man-UA), and guluronic acid (Gul-UA) were obtained from the Sigma-Aldrich Chemical Co. (St. Louis, MO, USA).

### 3.2. General Methods

Determination of CPTM-CP total sugars was conducted by the phenol–sulphuric acid method using glucose as a standard [7]. An HP-GPC system with tandem UltrahydrogelTM2000 and UltrahydrogelTM250 columns was used to analyze the molecular weight of CPTM-P1. A Fourier-transform IR spectrophotometer (Perkin-Elmer Corp., Waltham, MA, USA) was used to scan FTIR spectra. A Thermo ICS5000 (Thermo Fisher Scientific Corp., USA) ion chromatography system with a Dionex CarboPac PA20 (150 × 3.0 mm, 10 um) liquid chromatography column was used for HPLC-MS.

### 3.3. Extraction, Isolation, and Purification of CPTM-P1

**Figure 8:**
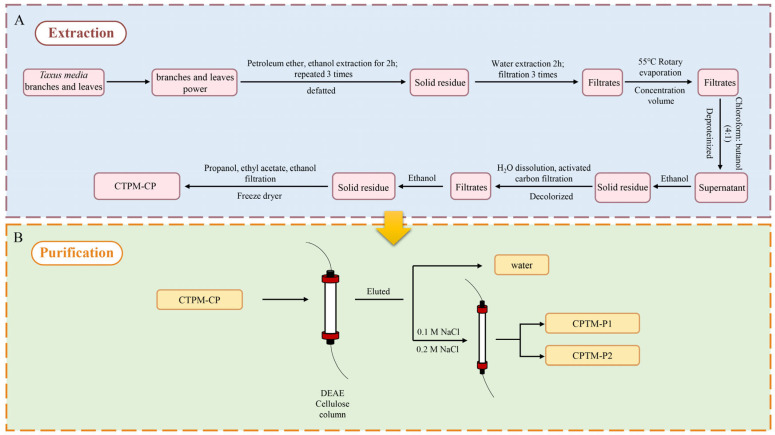
Extraction and purification flowchart of CPTM−P1 from *Taxus media*. (**A**) is the extraction flowchart, and (**B**) is the purification flowchart.

Three hundred grams of powdered *Taxus media* branches and leaves were initially treated with petroleum ether and 80% ethanol to remove fat-soluble substances, alcohol-soluble impurities, monosaccharides, and oligosaccharides. This was followed by triple water extractions to eliminate remaining impurities. Proteins were then removed using Sevag reagent (n-butanol: chloroform = 1:4). This procedure was repeated until the supernatant no longer exhibited absorption peaks at 260 and 280 nm, indicating complete protein removal, thereby allowing progression to the next phase. The extract was mixed with anhydrous ethanol and left to precipitate overnight at 4 °C. The resulting precipitate was collected, sequentially washed with ethanol, acetone, ethyl acetate, and anhydrous ethanol, then dissolved in distilled water, decolorized with activated charcoal, filtered, and finally collected. A freeze dryer was employed to produce the crude polysaccharide. A 1.000 g sample of CPTM-CP was dissolved in 40 mL of distilled water and filtered through a 0.45-micrometer membrane. The filtrate was then applied to a DEAE-cellulose column (2.6 × 30 cm). The column underwent washing with water and two concentrations of sodium chloride, maintaining a flow rate of 1 mL/min at room temperature. After washing, two distinct polysaccharide fractions, CPTM-P1 and CPTM-P2, were isolated via phased elution. The DEAE profile was analyzed using the phenol-sulfuric acid method. The entire extraction and purification process is detailed in Figure 8.

### 3.4. Analysis of Gel Permeation Chromatograph (GPC)

For GPC analysis, a total of 5.0 mg of the sample was dissolved in ddH_2_O for GPC analysis. The analysis was conducted using a Waters ultra-hydrophilic linear column (300 × 7.8 mm) at room temperature, with 0.2 M phosphoric acid buffer for elution at a flow rate of 0.70 mL/min. The standard reference for calibration ranged from dextran molecules of 2500 Da to 2,000,000 Da [43].

### 3.5. Monosaccharide Analysis

A 5 mg sample of the CPTM-P1 polysaccharide was hydrolyzed with trifluoroace-tic acid (TFA) at 121 °C for two hours. Post-hydrolysis, the sample was washed with methanol two to three times, dried under a stream of nitrogen gas, and reconstituted in sterile water for analysis. Ion chromatography was used to analyze the monosaccharide fraction after adding sterile water to the dry powder. The liquid chromatography column used was a Dionex™ CarboPac™ PA20 (150 × 3.0 mm, 10 um). The injection volume was 5 uL. The chromatographic conditions were: mobile phase A (0.1 M NaOH), mobile phase B (0.1 M NaOH, 0.2 M NaAc), flow rate 0.5 mL/min; column temperature 30 °C; elution gradient: 0 min A-phase/B-phase (95:5 *v*/*v*), 30 min A-phase/B-phase (80:20 *v*/*v*), 30.1 min A-phase/B-phase (60:40 *v*/*v*), 45 min A-phase/B-phase (30.1 min A-phase/B-phase). (30 min A-phase/B-phase, 80:20 *v*/*v*, 30.1 min A-phase/B-phase, 45 min A-phase/B-phase, 45.1 min A-phase/B-phase, 60 min A-phase/B-phase, 95:5 *v*/*v*). The monosaccharide standards, including fucose (Fuc), arabinose (Ara), rhamnose (Rha), galactose (Gal), glucose (Glc), xylose (Xyl), mannose (Man), fructose (Fru), ribose (Rib), galacturonic acid (Gal-UA), glucuronic acid (Glc-UA), mannuronic acid (Man-UA), and guluronic acid (Gul-UA), were obtained from the Sigma-Aldrich Chemical Co. (St. Louis, MO, USA). The identification and quantification of different sugars were achieved, respectively, by comparing the retention time and peak area with those of monosaccharide standards.

### 3.6. Fourier-Transform Infrared (FT-IR) Analysis

For the FT-IR analysis, purified CPTM-P1 was mixed with potassium bromide (KBr) powders in a 1:20 weight ratio (*w*/*w*), ground thoroughly, and pressed into thin pellets. These were then analyzed using a Fourier Transform Infrared Spectrophotometer (Perkin-Elmer Corp., USA) across a frequency range of 500–4000 cm^−1^ to record the FT-IR spectra.

### 3.7. Nuclear Magnetic Resonance Spectroscopy (NMR) Analysis

NMR spectroscopy, known for its high-resolution capability in elucidating molecular structures, was utilized to examine CPTM-P1 in detail. Fifty milligrams of CPTM-P1 were dissolved in 0.5 mL of deuterium oxide (D_2_O) and lyophilized three times. The lyophilized sample was redissolved in 0.5 mL of D_2_O and analyzed in an NMR tube using a Bruker AVANCE IIIHD 600 spectrometer (Bruker, Rheinstetten, Germany) at 600 MHz for both ^1^H-NMR and ^13^C-NMR at 25 °C. Acetone served as the internal standard for ¹³C NMR, with chemical shifts reported in parts per million (ppm).

### 3.8. Scanning Electron Microscope (SEM) Analysis

For SEM analysis, the dried CPTM-P1 polysaccharide underwent ion sputtering to ensure electron conductivity. The coated sample was examined under a scanning electron microscope set to an acceleration voltage of 15 kV and magnifications ranging from 1000 to 10,000×. This SEM analysis facilitated a detailed observation and documentation of the poly-saccharide’s surface characteristics.

### 3.9. Measurement of Anti-Inflammatory Activity In Vitro

#### 3.9.1. Culture of RAW264.7 Cells

RAW264.7 cells were sourced from Procell Life Science & Technology (Wuhan, China). The cells were cultured in Dulbecco’s modified Eagle’s medium (DMEM, Gibco, Beijing, China) supplemented with 10% (*v*/*v*) fetal bovine serum (FBS, Biological Industries, Bioind, Kibbutz Beit, Israel), 100 U/mL penicillin, and 100 μg/mL streptomycin. The cell culture was maintained at 37 °C in a 5% CO_2_ atmosphere.

#### 3.9.2. RAW264.7 Cell Proliferation and Phagocytic Capacity Analysis

RAW264.7 cells in the logarithmic growth phase were diluted and plated in 96-well plates at a density of 6 × 10^4^ cells per well, with 100 µL of cell suspension per well. The blank control received fresh medium only. Each concentration gradient had triplicate wells. After 24 h of incubation at 37 °C in 5% CO_2_, the supernatant was discarded, and wells were treated with 100 µL of varying concentrations (0.25, 0.5, 1, 2, and 4 mg/mL) of the polysaccharide CPTM-P1 solution, while controls received fresh medium. Following an additional 24-hour incubation, cell proliferation was assessed using the MTT assay, with optical density (OD) measured at 490 nm. For phagocytic capacity analysis, cells were washed with phosphate-buffered saline (PBS) and stained with 0.075% neutral red for 1 h. After staining, wells were rinsed with PBS and lysed with an ethanol acetate solution (1:1 *v*/*v*), left overnight at room temperature. The OD was measured at 492 nm to evaluate the cells’ phagocytic capacity.

#### 3.9.3. Analysis of RAW264.7 Cell’s Secretion Ability of TNF-α, IL-1β, IL-6, and NO

Macrophages, maintained in a favorable growth condition at a density of 6 × 10^4^ cells/mL, were subjected to incubation with 0.5 μg/mL of lipopolysaccharide (LPS) along with various concentrations of CPTM-P1 (0.25, 0.5, 1, 2, and 4 μg/mL) as a positive control. This incubation process took place at 37 °C for a duration of 24 h. The culture supernatant was collected, and the levels of TNF-α, IL-1β, and IL-6 were determined using an enzyme-linked immunosorbent assay (ELISA). The content of nitric oxide (NO) in the RAW264.7 cell culture supernatant was assessed using the Griess method.

### 3.10. Removal of Endotoxin

To mitigate the potential impact of endotoxin contamination on the immunomodulatory activity of CPTM-P1, Affi-Prep Polymyxin Matrix (BIO-RAD, Hercules, CA, USA) was employed to remove potential endotoxin contaminants in CPTM-P1. Initially, 1 mL of Affi-Prep Polymyxin Matrix was packed into a Bio-spin column (BIO-RAD) and centrifuged at 300× *g* for 3 min. Subsequently, 1 mL of CPTM-P1 (1 mg/mL), LPS (0.5 μg/mL), or a mixture of CPTM-P1 (1 mg/mL) and LPS (0.5 μg/mL) was added. After overnight incubation at 4 °C, the liquid eluted under the same centrifugation conditions was collected from the recovered chromatography column, which was performed in three biological replicates.

### 3.11. Statistical Analysis

Statistical analysis was performed using IBM SPSS version 27.0. Each experiment was independently conducted three times, with results expressed as mean ± standard deviation. Differences between groups were evaluated using a one-way analysis of variance (ANOVA), with statistical significance set at *p* < 0.05 or *p* < 0.01.

## 4. Conclusions

In this study, we successfully isolated and purified a *Taxus media* polysaccharide, CPTM-P1, and meticulously characterized its molecular attributes (Figure 1). Employing high-performance gel permeation chromatography (HP-GPC) and high-performance liquid chromatography-mass spectrometry (HPLC-MS), we ascertained CPTM-P1’s molecular weight to be 968.7 kDa, with a composition rich in monosaccharides like galactose (Gal), galacturonic acid (Gal-UA), and arabinose (Ara). Nuclear magnetic resonance (NMR) spectroscopy further detailed the structural intricacies of CPTM-P1’s sugar chains. The pronounced immunomodulatory activity of CPTM-P1 was highlighted by its capacity to enhance the secretion of nitric oxide (NO), tumor necrosis factor-alpha (TNF-α), interleukin-1β (IL-1β), and interleukin-6 (IL-6), without dampening macrophage function. This underscores CPTM-P1’s favorable safety profile. Ultimately, CPTM-P1 emerges as a promising candidate for immuno-modulatory applications, boasting low toxicity and significant potential for use in functional foods or pharmaceutical products.

## Figures and Tables

**Table 1:** Monosaccharide composition of CPTM−P1.

Monosaccharide	Content (mg/g)	Percentage (%)
Gal	36.42	30.53
Ara	26.24	22.00
Rha	14.23	5.63
Glc	13.92	11.67
Gal-UA	27.42	11.93
Xyl	2.01	1.69
Fuc	1.47	8.5
Man	10.14	1.23
Rib	1.4	5.63
Glc-UA	6.72	1.17

**Table 2:** Chemical shifts in the ^1^H NMR and ^13^C NMR of CPTM−P1.

Glycosyl Residues	Chemical Shift H/C (ppm)					
	H_1_/C_1_	H_2_/C_2_	H_3_/C_3_	H_4_/C_4_	H_5_/C_5_	H_6_/C_6_
α-D-Galp-(1→	5.12/99.52	3.80/68.68	3.89/69.37	4.024/70.36	4.12/70.69	3.71/61.36
→6)-α-D-Glcp-(1→	5.12/99.52	3.62/76.71	3.71/73.72	3.55/70.36	3.96/70.69	4.07/67.01
→1-(-α-L-Rhap-(2→	5.30/95.47	4.14/76.71	3.96/69.37	3.40/73.37	3.80/68.88	1.14/16.78
→1)-α-L-Araf-(5→	5.12/107.54	4.14/78.74	4.07/76.71	4.18/82.08	3.96/68.88	-
α-D-Manp-(1→	5.12/99.52	4.07/70.36	3.87/70.69	3.71/68.68	3.78/73.72	4.02/61.33
→2)-α-D-Manp-(1→	5.30/99.52	4.07/78.74	4.00/70.69	3.67/68.68	3.71/73.72	3.80/61.33
→4)-α-GalpA-(1→	4.99/99.52	3.89/70.69	4.12/73.37	4.36/78.74	4.68/73.77	177.848
α-D-GlcAp-(-1→	5.30/99.52	3.62/73.72	3.71/76.71	3.55/73.72	4.02/73.37	-

## Data Availability

Dataset available on request from the authors.
